# Effects of Advective-Diffusive Transport of Multiple Chemoattractants on Motility of Engineered Chemosensory Particles in Fluidic Environments

**DOI:** 10.3390/e21050465

**Published:** 2019-05-04

**Authors:** Danielle King, Hakan Başağaoğlu, Hoa Nguyen, Frank Healy, Melissa Whitman, Sauro Succi

**Affiliations:** 1 Department of Mathematics, The University of Texas, Austin, TX 78712-1202, USA; 2 Mechanical Engineering Division, Southwest Research Institute, San Antonio, TX 78238-5166, USA; 3 Department of Mathematics, Trinity University, One Trinity Place, San Antonio, TX 78212-7200, USA; 4 Department of Biology, Trinity University, One Trinity Place, San Antonio, TX 78212-7200, USA; 5 Fondazione Istituto Italiano di Tecnologia, Center for Life Nanoscience at la Sapienza, vle Regina Margherita, 00165 Rome, Italy; 6 Istituto Applicazioni del Calcolo, Via dei Taurini 19, 00185 Roma, Italy

**Keywords:** chemotaxis, engineered chemosensory particle, multiple chemoattractants, particle-fluid hydrodynamics, multiscale numerical model

## Abstract

Motility behavior of an engineered chemosensory particle (ECP) in fluidic environments is driven by its responses to chemical stimuli. One of the challenges to understanding such behaviors lies in tracking changes in chemical signal gradients of chemoattractants and ECP-fluid dynamics as the fluid is continuously disturbed by ECP motion. To address this challenge, we introduce a new multiscale numerical model to simulate chemotactic swimming of an ECP in confined fluidic environments by accounting for motility-induced disturbances in spatiotemporal chemoattractant distributions. The model accommodates advective-diffusive transport of unmixed chemoattractants, ECP-fluid hydrodynamics at the ECP-fluid interface, and spatiotemporal disturbances in the chemoattractant concentrations due to particle motion. Demonstrative simulations are presented with an ECP, mimicking *Escherichia coli* (*E. coli*) chemotaxis, released into initially quiescent fluids with different source configurations of the chemoattractants *N*-methyl-L-aspartate and L-serine. Simulations demonstrate that initial distributions and temporal evolution of chemoattractants and their release modes (instantaneous vs. continuous, point source vs. distributed) dictate time histories of chemotactic motility of an ECP. Chemotactic motility is shown to be largely determined by spatiotemporal variation in chemoattractant concentration gradients due to transient disturbances imposed by ECP-fluid hydrodynamics, an observation not captured in previous numerical studies that relied on static chemoattractant concentration fields.

## 1. Introduction

Intellectual and technological advances in a variety of fields continue to refine our understanding of the principles and potential applications of nanorobotic systems. Of great interest in this area is the understanding and development of control systems through which nanorobotic devices or bacterial biohybrids carrying a payload can be effectively directed to a specified target. Work in this field holds particular promise in applications relevant to health and biomedicine [1,2,3,4,5]. In addition to considerations of cargo and payload storage design, successful nanorobotics-based therapies must rely on a clear understanding of the structure and function of actuators, sensors, and power sources that govern the devices’ abilities to interact with their fluidic environments [6]. While the fluidic environment poses navigational challenges to device design in each of these areas [7], natural biological systems, which have undergone adaptation and evolutionary selection for optimized solutions to these issues, have provided insights and inspiration [8,9,10,11,12]. Indeed, bacterial cells have been engineered to target specific locations in animal systems, most notably cancer tissue [2,13,14,15].

A variety of actuation systems have been explored, including magnetic and acoustic fields, light and chemical energy [5]. While each of these actuating systems is characterized by distinct design features, advantages and limitations, they all share in common the fact that the engineered device operates in a fluidic environment. Therefore, it is essential to computationally and experimentally investigate how various fluidic environments influence the motility of particles, with the guiding principle that an understanding of actuation mechanics as well as particle-fluid dynamics will provide valuable insights into the design properties and behaviors of engineered devices in fluids. As such, biomimetics of the flagellar chemosensing bacterium *Escherichia coli* (*E. coli*), whose motility in fluids is driven by sensing chemical gradients and transformation of electrochemical energy into motion [16], could lead to the enhanced design of autonomous chemosensory structures with diverse applications in a variety of fluidic environments.

**Figure 1:**
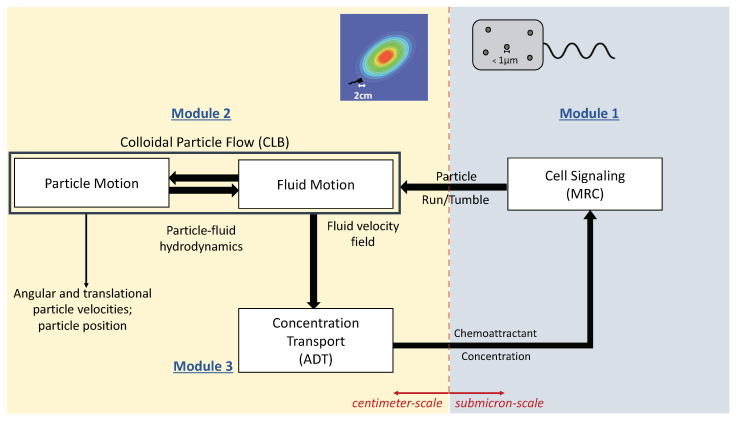
Coupling of the modules of the multiscale MRC-CLB-ADT model and information exchanges among the modules.

In this paper, we present a novel multiscale numerical model to simulate chemotactic behavior of an engineered chemosensory particle (ECP) swimming in a fluidic environment (Figure 1). The model is built through the dynamic coupling of three modular components involving (i) ECP chemical attractant signal sensing and swimming response based on the chemotaxis adaptive phosphorelay circuit that governs the operation of the *E. coli* flagellar motor (MRC module); (ii) chemotactic ECP hydrodynamic interactions with the bulk fluid (CLB module); and (iii) chemical substrate transport phenomena in a fluidic environment, validated against a 2D benchmark problem and described in this paper (ADT module). The first module (MRC) simulates submicron−scale cell signaling processes that determine the run and tumble biased random walk behavior of the ECP based on the chemical environment. The second and third modules (CLB and ADT) simulate particle-fluid hydrodynamics and spatiotemporal variations in the fluid velocity and substrate concentration at the cm-scale. Hence, the new model is a coupled multiscale numerical model that simulates the transformation of chemical energy by an ECP to mechanical energy as it swims through fluidic environments containing concentration gradients of chemoattractants. This is accomplished by accommodating dynamic changes in spatiotemporal distributions in the fluid velocity and concentration fields due to ECP motion. Details of each of these module components comprising the multiscale model are described below.

We recently developed multiscale computational models for *E. coli* chemotactic sensing by coupling the Monod-Wyman-Changeux mixed chemosensory receptor cluster model, known as RapidCell (RC) [17], with the method of regularized Stokeslets [18] or the colloidal lattice Boltzmann (CLB) models [19,20]. These models were used to simulate motility of chemosensory particles in confined fluidic environments with externally imposed chemoattractant gradients [21,22]. In this paper, we extend the coupled RC-CLB model to simultaneously simulate advective-diffusive transport (ADT) of two unmixed chemoattractants, ECP-fluid hydrodynamics, and disturbances in spatiotemporal distributions of chemoattractants due to particle motion. We equip the ECP with the well-established model of chemical stimulus sensing circuitry of *E. coli*, which confers on the ECP the ability to detect and respond to gradients of chemoattractant compounds in the fluidic environment. We refer to our upgraded model as MRC-CLB-ADT, corresponding to the modified RapidCell (MRC)-colloidal lattice Boltzmann (CLB)-advective-diffusive chemoattractant transport (ADT) model. The MRC-CLB-ADT model is used in this paper to follow the position and swimming trajectories of an ECP in fluidic environments in which two unmixed chemoattractants are introduced on opposite sides of a confined flow domain. We also utilize the model to investigate how the residence times of the ECP are affected by chemoattractant release mode (instantaneous vs. continuous, point source vs. distributed) as concentration and fluid velocity fields are altered through ECP swimming behavior.

In the following sections, we first describe the mathematical formulation of the new coupled MRC-CLB-ADT model to simulate the motility of ECPs in two-dimensional (2D) fluidic environments in response to spatiotemporal variations in concentrations of the amino acid chemoattractants *N*-methyl-L-aspartate (MeAsp) and L-serine (Ser). The selection of these amino acids is based on their frequent use in experimental studies of *E. coli* chemotaxis mechanics and chemoreceptor biochemistry [16]. Using this modeling framework, we present and discuss the results from simulations with increasing levels of complexity and realism. The first set of simulations is performed using imposed temporally-invariant, but spatially-variant concentration fields. In these simulations, chemoattractant distributions are presumably not affected by ECP-fluid hydrodynamics, and are used as benchmark cases. A second more realistic set of simulations incorporates the effects of ECP motility in the fluidic environment on the chemoattractant distributions. Through the incorporation of a dynamically-evolving mixed chemical environment, the MRC-CLB-ADT model simulations reveal critical roles of the ECP-fluid hydrodynamics on the chemosensory particle motility not previously recognized in static concentration field-based models.

## 2. Mathematical Framework of the MRC-CLB-ADT Model

A mathematical description of main submodels (MRC, CLB, and ADT) and the optional submodel (static chemoattractant concentration fields) of the MRC-CLB-ADT model and their coupling are provided in this section. Numerical validations of the CLB and ADT modules are also discussed in this section.

### 2.1. Module 1. Modified RapidCell (MRC) Model for Particle Chemosensing in Two Chemoattractant Fields

**Figure 2:**
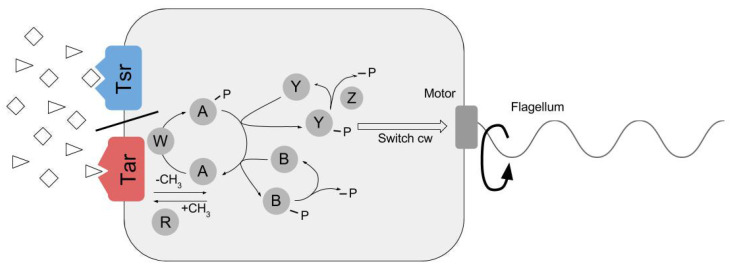
Chemotactic signaling by Tar and Tsr MCPs in *E. coli*. Chemoattractants such as *N*-methyl-L-aspartate and L-serine (represented by triangle and diamond shapes) are sensed by Tar and Tsr MCPs, respectively, and binding results in signal transduction across the cell membrane to a phosphorelay response circuit. Phosphoryl group (P) transfer to Che proteins controls direction of rotation of flagellar motor and MCP methylation-dependent adaptive response. Default flagellar rotation is counterclockwise, causing cell to run; switching to clockwise rotation results in reorientation of cell through tumbling motion due to flagellar unbundling. Circled letters represent Che proteins described in text, e.g., A represents CheA, Y represents CheY, etc.

We modified the RapidCell (RC) model to simulate chemotactic motility of the ECP with the premise that the ECP mimics *E. coli* chemotaxis in the presence of two unmixed chemoattractants. The RC model [17] was originally developed to simulate flagellar bacterial chemotaxis in an environment with a spatiotemporally varying concentration gradient of a single chemoattractant, and performs two tasks: chemoattractant signal processing by the methyl-accepting chemotaxis protein (MCP) sensory lattice and adaptive feedback response of the sensory array [16,23,24,25]. Signal processing by the cell occurs through interactions between chemoattractant-activated chemoreceptors, CheA kinase, and other regulators including CheY, CheR, CheB, and CheZ (Figure 2). Chemoattractant-receptor interactions regulate the autophosphorylation activity of CheA [24]. CheA-P phosphoryl transfer activity may then result in switching of direction of motor rotation through CheY-P [26] or adjustment of adaptive response through MCP methyl group hydrolysis by CheB-P [27].

The RC model was modified to account for the presence of two unmixed chemoattractants using the total free energy differences in [28]. The effect of the total free energy differences between ‘on’ and ‘off’ states for two receptors sensing two chemoeffectors is described as

(1) $$F=N\left[h\left(m\right)+\nu_{a}\ln\left(\frac{1+\left[MeAsp\right]/K_{a}^{off}}{1+\left[MeAsp\right]/K_{a}^{on}}\right)+\nu_{s}\ln\left(\frac{1+\left[Ser\right]/K_{s}^{off}}{1+\left[Ser\right]/K_{s}^{on}}\right)\right],$$

where *N* is the number of chemoreceptors in the receptor cluster. [MeAsp] and [Ser] are the extracellular chemoattractant MeAsp and Ser concentrations, respectively [28]. The binding of MeAsp by Tar is given in the modified total free energy by $\nu_{a}$ and the binding of Ser by Tsr is represented in the modified total free energy by $\nu_{s}$. The offset energy, h(m) is given by 1−m/2 where *m* is receptor methylation defined in Equation (4). The dissociation constant for the chemoattractant in the ‘on’ or ‘off’ state is specified as $K_{r}^{on/off}$(r=a,s).

The total free energy differences *F* from Equation (1) are used in the RC model to compute the receptor methylation (*m*) and the basal motor bias (mb)

(2) $$\begin{array}{c} A_{c}=\frac{1}{1+e^{F}} \end{array}$$

(3) $$\begin{array}{c} \left[\mathrm{CheY}-P\right]=3\frac{k_{Y}k_{s}A_{c}}{k_{Y}k_{s}A_{c}+k_{Z}{\left[\mathrm{CheZ}\right]}_{tot}+\gamma_{Y}}, \end{array}$$

(4) $$\begin{array}{c} \frac{dm}{dt}=a\left(1-A_{c}\right)\left[\mathrm{CheR}\right]-bA_{c}\left[\mathrm{CheB}\right], \end{array}$$

(5) $$\begin{array}{c} mb=(1+\left(1/mb_{0}-1\right){\left({\left[\mathrm{CheY}-P\right]}^{H}\right)}^{-1}, \end{array}$$

where $A_{c}$ is the probability of the cluster activity, CheY-P is the concentration of phosphorylated CheY, $k_{Y}$, $k_{Z}$ and $\gamma_{Y}$ are the rate constants, $k_{s}$ is the scaling coefficient, ${\left[\mathrm{CheZ}\right]}_{tot}$ is the total CheZ concentration, *a* and *b* are the methylation scaling factors, *m* is receptor methylation, mb is the motor bias, $mb_{0}$ is the basal motor bias, and *H* is the motor Hill coefficient. In RC model simulations, the initial methylation state of the receptor cluster is obtained from a steady-state methylation level associated with the initial chemoattractant concentration bound to the chemoreceptor cluster. The methylation is then updated by solving Equation (4) using the forward Euler finite difference method. Motor bias values may range from 0 to 1 with higher values corresponding to a greater likelihood of running motion of bacteria. To determine whether a particle will run or tumble, a uniform random number ξ is generated between 0 and 1; and if ξ<mb, the particle runs; otherwise, it tumbles. A complete list and description of the parameters, variables, and functions in Module Section 2.1 are provided in Table A1 and Table A3 in the Appendix A.4.1.

Our modification to the RC model provides a framework for relating the time history of multiple chemoattractant concentration sensing events at the particle’s chemosensory array to the run-tumble probability output and is called the modified RapidCell model (MRC).

#### 2.1.1. Static (Time-Invariant) Concentration Fields

If externally-computed time-invariant radial concentration gradients are used for MeAsp and Ser in numerical simulations, the concentration gradients are described by [28]

$$\begin{array}{r} \left[MeAsp\right]=\omega \left[C_{a0}+\exp\left(-\sqrt{{\left(x+x_{a}\right)}^{2}+y^{2}}\right)\right]\\ \left[Ser\right]=\nu \left[C_{s0}+\exp\left(-\sqrt{{\left(x+x_{s}\right)}^{2}+y^{2}}\right)\right], \end{array}$$

where $C_{a0}$ and $C_{s0}$ are the minimum chemoattractant concentrations for MeAsp and Ser, respectively. *x* and *y* are the horizontal and vertical coordinates, and ω and ν are scaling parameters for MeAsp and Ser gradients, respectively. The parameters $\left(x_{a},y_{a}\right)$ and $\left(x_{s},y_{s}\right)$ describe the location of the maximum MeAsp and Ser concentrations in a 2D square domain, described as $\left(x,y\right)=\{x,y\in R:\left[-L^{*},L^{*}\right]\}$, in which $L^{*}$ is the domain length [28]. The imposed chemoattractant concentration gradients of MeAsp and Ser are scaled, using a scaling parameter of *r*, for larger domains in RC-CLB simulations as follows

(6) $$\begin{array}{c} \left[MeAsp\right]=\omega r\left[C_{a0}+\exp\left(\frac{-\sqrt{{\left(x+x_{a}\right)}^{2}+{\left(y+y_{a}\right)}^{2}}}{r}\right)\right], \end{array}$$

(7) $$\begin{array}{c} \left[Ser\right]=\nu r\left[C_{s0}+\exp\left(\frac{-\sqrt{{\left(x+x_{s}\right)}^{2}+{\left(y+y_{s}\right)}^{2}}}{r}\right)\right]. \end{array}$$

#### 2.1.2. Dynamic (Time-Variant) Concentration Fields

In reality, the MeAsp and Ser concentrations change according to their own diffusion process while being advected by the fluid flow and the ECP motion as time goes on. Therefore, the concentration fields cannot be externally computed and independent of time as in the ideal case of Section 2.1.1. At each time step the fluid-ECP interactions need to be taken into account so that the distributions of MeAsp and Ser concentrations can be updated properly. Section 2.2 presents how the fluid-ECP interactions are modeled. Section 2.3 shows how the resultant fluid velocities from Section 2.2 affect the transport of MeAsp and Ser.

### 2.2. Module 2. Colloidal Lattice Boltzmann (CLB) Model for Particle-Fluid Interactions

The CLB model has two submodules, (i) fluid flow submodule and (ii) particle flow submodule, The former calculates the local changes in the fluid velocity field in response to ECP motion. The latter subsequently updates angular and translational velocities of an ECP in the disturbed fluid velocity field. These two new submodules operate sequentially in each time step and calculations are based on momentum exchanges between a motile ECP and the bulk fluid across the ECP’s surface. In the fluid flow module, the fluid flow is governed by the Navier-Stokes equation and it is solved using the lattice-Boltzmann method (LBM) [29]. In the particle flow module, the angular and translational velocities of an ECP are computed based on Newton’s equation of motion. Mathematical details of these two submodels are presented next.

#### 2.2.1. Fluid Flow Submodule (FFS)

In the fluid flow submodule of the CLB model, the mesodynamics of the transient, weakly compressible, Newtonian fluid flow is described by a single relaxation time [30].

(8) $$f_{i}\left(r+e_{i}▵t,t+▵t\right)-f_{i}\left(r,t\right)=\frac{▵t}{\tau }\left[f_{i}^{eq}\left(r,t\right)-f_{i}\left(r,t\right)\right],$$

where $f_{i}\left(r,t\right)$ is the complete set of population density of discrete velocities $e_{i}$ at position r and discrete time *t* with a time increment of ▵t, and τ is the relaxation parameter. The left-hand side of Equation (8) describes the streaming of populations from a lattice node r to the closest neighbor in the direction $e_{i}$. The right-hand side of Equation (8) represents the local collision process. $f_{i}^{eq}$ in Equation (8) is the discrete equilibrium Maxwell-Boltzmann distribution approximated by the low Mach number mass and momentum conserving expansion [31]

(9) $$f_{i}^{eq}=\omega_{i}\rho \left(1+\frac{e_{i}\cdot u}{c_{s}^{2}}+\frac{{\left(e_{i}\cdot u\right)}^{2}}{2c_{s}^{4}}-\frac{u\cdot u}{2c_{s}^{2}}\right),$$

where $\omega_{i}$ is the weight associated with $e_{i}$, $c_{s}$ is the speed of sound, $c_{s}=▵x/\sqrt{3}▵t$, and ▵x is the lattice spacing. The local fluid density, ρ, and velocity, u, at the lattice nodes are given by

(10) $$\rho =\sum_{i}f_{i},\;\;\rho u=\sum_{i}f_{i}e_{i}+\tau \rho g,$$

where g is the acceleration due to external forces [32]. In Equation (10), $\rho g=▵P/L^{*}$, and hence, ρg specifies the pressure differential across the fludic domain with the length of $L^{*}$. If the fluidic domain is stagnant, then g=0. Otherwise, the flow strength across the fluidic domain can be specified by ρg.

**Figure 3:**
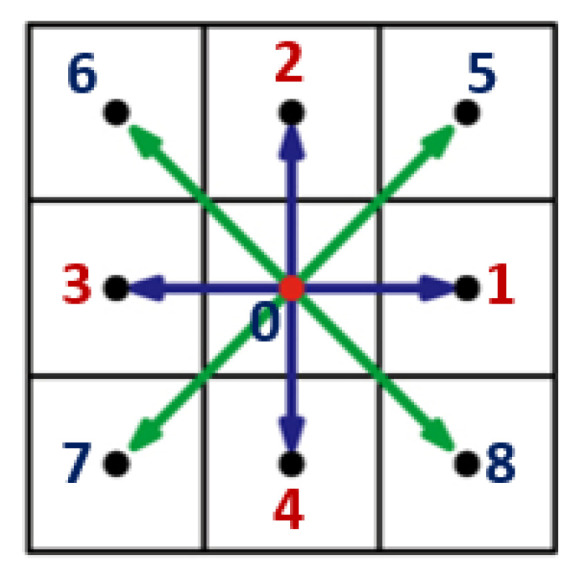
D2Q9 (two-dimensional nine velocity vector) lattice geometry. The vector basis set for the D2Q9 model consists of a null vector (rest population), which improves the stability of the algorithm, four off-diagonal vectors of length unity directed towards the nearest neighbor nodes, and four diagonal vectors of length $\sqrt{2}$ directed toward the next-nearest neighboring nodes.

Through the Chapman-Enskog approach [29], in the limit of small Knudsen number for weakly compressible fluids ($▵\rho /\rho ∼M^{2}∼1\times 10^{-4}$, where *M* is the Mach number), the LB method for single-phase fluid flow recovers the Navier-Stokes equations

(11) $$\nabla \cdot u∼0,\;\;\partial_{t}u+\left(u\cdot \nabla \right)u=-\frac{\nabla P}{\rho }+\tilde{\nu }\nabla^{2}u+g.$$

where $\tilde{\nu }$ is the kinematic viscosity of the fluid. Pressure is computed via the equation of state for an ideal gas, $P=c_{s}^{2}\rho$. A commonly used D2Q9 (two-dimensional nine velocity vector) lattice geometry (Figure 3), which ensures fourth-order lattice isometry, was adopted in LB simulations in this paper.

For which unit discrete velocities $e_{i}$ at each lattice node are defined as

(12) $$e=\left[\begin{array}{ccccccccc} 0 & 1 & 0 & -1 & 0 & 1 & -1 & -1 & 1\\ 0 & 0 & 1 & 0 & -1 & 1 & 1 & -1 & -1 \end{array}\right].$$

The first column vector of e in Equation (12) is the null vector corresponding to the rest population, the second through fifth column vectors correspond to the four vectors of length unity directed toward the nearest neighboring lattice nodes, and the sixth through ninth column vectors correspond to the four vectors of length $\sqrt{2}$ directed toward the next-nearest neighboring lattice nodes (Figure 3). Equation (8) recovers the Navier-Stokes equation for $\omega_{i}=4/9$ for the rest populations (i=0), $\omega_{i}=1/9$ for the off-diagonal populations $\left(i\in \left[1,4\right]\right)$, and $\omega_{i}=1/36$ and for the diagonal populations $\left(i\in \left[5,8\right]\right)$ in Equation (9) and $\tau =0.5+3\tilde{\nu }\left[▵t/{\left(▵x\right)}^{2}\right]$ in Equation (8), which are obtained through the Chapman-Enskog expansion [29]. Hence, τ in the LB method is determined by the kinematic viscosity of the fluid, $\tilde{\nu }$. Numerical stability in fluid flow simulations was ensured by τ=0.8<1.0.

In each time-step in numerical simulations, $f_{i}$ and $f_{i}^{eq}$ were computed at each lattice node via Equations (8) and (9). $f_{i}$’s can be altered locally by ECP motion, which will be discussed in the next section. After $f_{i}$’s were computed, the fluid velocity and density at each lattice node were computed via Equation (10). In these simulations, the fluid was Newtonian; therefore, $\tilde{\nu }$, and hence, τ were constants. Moreover, ▵x and ▵t remained constant throughout simulations.

The LB method is second accurate in space and time. The LB model was preferred over classic Navier-Stokes solvers in this paper as the computationally demanding nonlinear convective term in the Navier-Stokes equation, $\left(u\cdot \nabla \right)u$, is replaced by a linear arithmetic streaming term (the left-hand side of Equation (8)) in the LB method. The streaming is exact and local mass and momentum conservations are accurate to machine round-off error [29,33].

#### 2.2.2. Particle Flow Submodule

**Figure 4:**
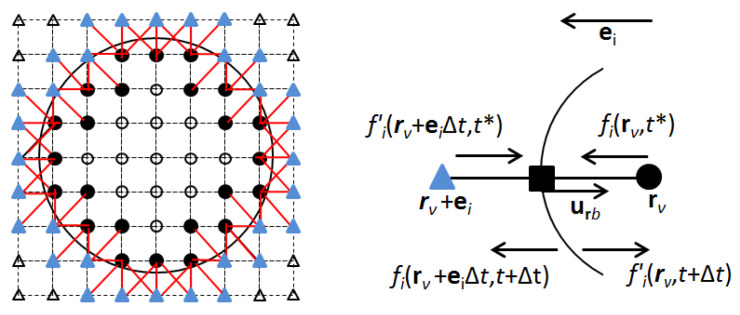
LB model representation of an ECP (left) and the momentum exchanges between the ECP and the fluid (right) [34,38,39]. Filled circles are the intra-particle virtual fluid nodes of ECP closest to its surface, filled triangles outside the ECP surface represent its extra-particle bulk fluid nodes, and the filled square represents the boundary node located half-way between the intra-particle node ($r_{v}$) and extra-particle node ($r_{v}+e_{i}▵t$). Hydrodynamic links along which the ECP exchanges momentum with the fluid are shown by red line segments.

The particle flow submodule of the CLB model [19,20], built on the LBM formulation by [34,35,36], was modified to simulate hydrodynamic interactions between an ECP (represented as a circular-cylindrical particle in the LBM) and the bulk fluid [21]. ECP-fluid hydrodynamic forces, $F_{r_{b}}$ at boundary nodes located halfway between the intra-particle lattice node, $r_{v}$, and extra-particle lattice node, $r_{v}+e_{i}$, are computed based on momentum exchanges between the ECP and surrounding fluid (Figure 4) [34,37]

(13) $$F_{r_{b}}=-2\left[f_{i}^{\prime }\left(r_{v}+e_{i}▵t,t^{*}\right)+\frac{\rho \omega_{i}}{c_{s}^{2}}\left(u_{r_{b}}\cdot e_{i}\right)\right]e_{i},$$

where $f_{i}^{\prime }$ is the population density in the $-e_{i}$ direction at the post-collision time $t^{*}$, and $u_{r_{b}}$ is the local particle velocity at the boundary node $r_{b}$. Equation (13) is related to the continuum-scale particle-fluid hydrodynamic force on the particle

(14) $$\sum_{r_{b}}F_{r_{b}}=m_{p}\left(u-U_{p}\right)/▵t,$$

where $m_{p}$ is the particle (ECP) mass and $U_{p}$ is the particle velocity. Steric interaction forces, $F_{r_{i}}$, between the particle and stationary solid zones are expressed in terms of two-body Lennard-Jones potentials [38] such that $F_{r_{i}}=-\psi {\left(\frac{|r_{i}|}{|r_{\mathrm{it}}|}\right)}^{-13}n$, where $|r_{i}|$ is the distance between a particle surface node and the stationary solid node located on channel walls or inline obstacles ($r_{i}=r_{pw}$); *p* is the ECP index; *w* is the wall or obstacles index; $|r_{it}|$ is the repulsive threshold distance; n is the unit vector along $r_{i}$; and ψ is the stiffness parameter used to adjust the repulsive strength between the particle and stationary solid zones. Then, the total force, $F_{T}$, on the ECP is

(15) $$F_{T}=\sum_{r_{b}}F_{r_{b}}+\sum_{r_{b}^{c,u}}F_{r_{b}^{c,u}}+\sum_{|r_{pw}|\leq |r_{it}|}F_{r_{pw}}+F_{run},$$

where $F_{r_{b}^{c,u}}=\pm \rho \left(u_{r_{b}^{c,u}}-U_{p}\right)/▵t$ is the force induced by covered, $r_{b}^{c}$, and uncovered, $r_{b}^{u}$, lattice nodes due to particle motion [35,37,39]. The force associated with the running motion of the ECP is defined as $F_{run}=\frac{\left(r_{cl}-r_{c}\right)}{|r_{cl}-r_{c}|}f_{m}$, where $r_{cl}$ is the location of the receptor cluster, $r_{c}$ is the position of the particle centroid, and $f_{m}$ is the force strength. $r_{cl}$ is placed on the boundary nodes. The total torque on the ECP, $T_{T}$, is defined as

(16) $$T_{T}=\sum_{r_{b}}\left(r_{b}-r_{c}\right)\times F_{r_{b}}+\sum_{r_{b}^{c,u}}\left(r_{b}^{c,u}-r_{c}\right)\times F_{r_{b}^{c,u}}+\sum_{|r_{\mathrm{pw}}|\leq |r_{\mathrm{it}}|}\left(r_{w}-r_{c}\right)\times F_{r_{pw}}+T_{tumble}.$$

Torque due to the ECP tumbling motion as it seeks the highest chemoattractant gradients, $T_{tumble}$, is

(17) $$T_{tumble}\left(t+▵t\right)=\frac{\Upsilon }{▵t}\left(\frac{▵\theta }{▵t}-\Omega_{tumble}\left(t\right)\right),$$

where Υ is the time-scaling factor associated with the angular rotation of the ECP due to its tumbling motion. This is different from the time-scale associated with its translation velocity resulting from running motion. $\Omega_{tumble}\left(t\right)$ is the ECP’s angular velocity due to particle torque resulting from its tumbling state at time *t* and is defined as $\Omega_{tumble}\left(t+\Delta t\right)=\Omega_{tumble}\left(t\right)+▵tT_{tumble}\left(t+▵t\right)/I_{p}$, where $I_{p}$ is the moment of inertia of the ECP ($I_{p}=m_{p}{r_{p}}^{2}/2$, in which $r_{p}$ is the radius of the circular ECP) and $\Omega_{tumble}\left(t=0\right)=0$. The angular rotation, ▵θ, of the ECP associated with its tumbling motion over ▵t is computed by ▵θ=2π(φ−0.5) where φ is a uniformly distributed random number between 0 and 1. The translational velocity, $U_{p}$, and the angular velocity of the ECP, $\Omega_{p}$, are advanced in time according to the discretized forms of Newton’s equations of motion

(18) $$U_{p}\left(t+▵t\right)\equiv U_{p}\left(t\right)+\frac{▵t}{m_{p}}F_{T}\left(t\right)+\frac{▵t}{\rho_{p}}\left(\rho_{p}-\rho \right)g,$$

(19) $$\Omega_{p}\left(t+▵t\right)\equiv \Omega_{p}\left(t\right)+\frac{▵t}{I_{p}}T_{T}\left(t\right).$$

Local velocities at the boundary nodes are computed by

(20) $$u_{r_{b}}=U_{p}+\Omega_{p}\times \left(r_{b}-r_{c}\right).$$

The new position of the ECP is computed by

(21) $$r_{c}\left(t+▵t\right)=r_{c}\left(t\right)+U_{p}\left(t\right)▵t.$$

Then the position of the receptor cluster on the ECP surface is updated by

(22) $$r_{cl}\left(t+▵t\right)=r_{c}\left(t+▵t\right)+r_{p}\left\{\cos\left[\theta_{cl}\left(t+\Delta t\right)\right],\sin\left[\theta_{cl}\left(t+\Delta t\right)\right]\right\},$$

where $r_{p}$ is the particle radius and the rotational angle of the receptor cluster is defined as $\theta_{cl}\left(t+\Delta t\right)=\theta_{cl}\left(t\right)+\Omega_{p}\left(t+▵t\right)▵t$. Finally, the population densities at the intra-particle node, $r_{v}$, and the extra-particle node, $r_{v}+e_{i}▵t$, are updated to account for momentum-exchange between the ECP and bulk fluid via [34] (Figure 4)

(23) $$f_{i}^{\prime }\left(r_{v},t+▵t\right)=f_{i}\left(r_{v},t^{*}\right)-\frac{2\rho \omega_{i}}{c_{s}^{2}}\left(u_{r_{b}}\cdot e_{i}\right),$$

(24) $$f_{i}\left(r_{v}+e_{i}▵t,t+▵t\right)=f_{i}^{\prime }\left(r_{v}+e_{i}▵t,t^{*}\right)+\frac{2\rho \omega_{i}}{c_{s}^{2}}\left(u_{r_{b}}\cdot e_{i}\right).$$

In each time-step in numerical simulations, the total force, $F_{T}$, and the total torque, $T_{T}$, on the ECP were computed via Equations (15) and (16). The resultant translational and angular velocities of the ECP were calculated by Equations (18) and (19). Local velocities at the boundary nodes of the ECP and the new position of the ECP were computed next by Equations (20) and (21). The new location of the receptor cluster on the ECP’s surface, at which chemical sensing of chemoattractants was simulated via MRC, was then computed by Equation (22). Local disturbances in the immediate vicinity of the motile ECP altered population densities, $f_{i}$, in accordance with Equations (23) and (24). The altered $f_{i}$’s near the ECP’s surface were used to compute the new fluid velocity field via Equations (8) and (10).

The CLB model was validated in [36] against numerically-computed (via the finite-element method) settling trajectories of a circular-cylindrical particle in an initially quiescent fluid [40]. As reported in [36], the CLB model also closely predicted terminal velocities of spherical particles 5% and 10% denser than the bulk fluid [36] in particle settling experiments reported in [41].

### 2.3. Module 3. Advective-Diffusive Transport (ADT) Model for Chemoattractant Distributions

In the original RC model [17], the chemoattractant environment surrounding chemosensory particles was not fluid-based; therefore, static chemottractant concentrations were externally computed (as discussed in Section 2.1.1) and artificially imposed onto the fluidic domain. To overcome this shortcoming, a chemoattractant transport model, based on the LBM, was formulated in this paper to simulate spatiotemporal distributions of chemoattractants in the fluidic environment of the ECP by accommodating the effects of particle motion-induced disturbances in the flow and chemoattractant concentration fields. The advective-diffusive chemoattractant transport was solved using the LBM on a D2Q9 lattice [42,43]:

(25) $$g_{i}\left(r+e_{i}▵t,t+▵t\right)-g_{i}\left(r,t\right)=\frac{▵t}{\tau_{c}}\left[g_{i}^{eq}\left(r,t\right)-g_{i}\left(r,t\right)\right],$$

where $g_{i}\left(r,t\right)$ is the complete set of population density of discrete velocities $e_{i}$ associated with the chemoattractant concentration at position r and time *t* with a time increment of ▵t. $\tau_{c}$ is the relaxation parameter associated with the chemoattractant transport. In Equation (25), $g_{i}^{eq}$ is the local equilibrium for the chemoattractant transport process and is given by [43]

(26) $$g_{i}^{eq}=\omega_{i}C\left(1+\frac{e_{i}\cdot u}{c_{s}^{2}}+\frac{{\left(e_{i}\cdot u\right)}^{2}}{2c_{s}^{4}}-\frac{u\cdot u}{2c_{s}^{2}}\right).$$

The local chemoattractant concentration at each lattice node r is given by $C=\sum_{i}g_{i}$. Through the Chapman-Enskog expansion [43], Equations (25) and (26), recovers the continuum-scale transient advective-diffusive substrate transport equation,

(27) $$\frac{\partial C}{\partial t}+u\cdot \nabla C=D\nabla^{2}C,$$

where *C* is the chemoattractant concentration, *D* is the Fickian diffusion coefficient, and u is computed by Equation (10). Equation (25) is equivalent to Equation (27) for $\tau_{c}=0.5+3D\left[▵t/▵x^{2}\right]$. The ADT model was validated with a 2D benchmark problem (Appendix A.1) and its performance was tested with different flow simulations (Appendix A.2) in the Appendix A.

In each time-step in numerical simulations, $g_{i}$ and $g_{i}^{eq}$ were computed at each lattice node via Equations (25) and (26), using the fluid velocity computed at each lattice node by Equation (10). After $g_{i}$’s were computed, chemoattractant concentrations at each lattice node were calculated by $C=\sum_{i}g_{i}$. Computed chemoattractant concentrations were used by the MRC to determine the tumble or run motion of the ECP. Decision on the tumble or run motion of the ECP affected $F_{T}$ and $T_{T}$ in Equations (15) and (16), and hence, altered local $f_{i}$’s in the vicinity of the mobile ECP according to Equations (23) and (24). A complete list and description of variables and parameters used in the CLB and ADT modules are provided in Table A3.

In this paper, we adopted the Fickian diffusion process via Equation (27), following the earlier numerical work by [42,43], in which *D* is constant in space and time, so that the diffusion process is described by $D\nabla^{2}C$. However, future work, involving also experimental tasks, will explore the use of the Fokker-Planck equation instead to describe the diffusive processes with temporal dispersion rather than spatial dispersion [44,45,46,47] of chemoattractant transport in determining chemotactic motility of ECPs. The LB method has been shown to be capable of solving 2D Fokker–Planck equations with variable coefficients by [48].

### 2.4. Coupling of the Modules, MRC-CLB-ADT Model

The MRC and CLB models are coupled via Equations (15), (16), and (22) [21]. Although each ECP is subject to its own signal processing and chemotactic swimming behavior, as described by Equations (1)–(5), their interactions with the bulk fluid and stationary solid zones are accounted for by particle-fluid hydrodynamic forces and particle-wall steric interaction forces in Equation (15). If the concentration fields for MeAsp and Ser are externally computed and imposed onto the fluidic domain using Equations (6) and (7), the coupled MRC-CLB model may be used to describe the transient behavior of ECPs without using the ADT model. Section 3.1 presents simulation results obtained by using such static concentration gradients.

**Figure 5:**
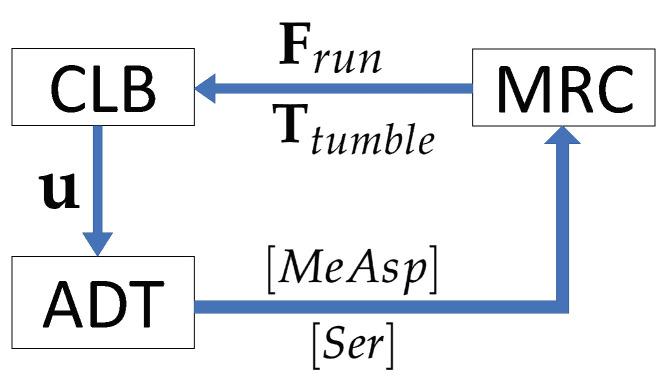
Data exchanges between submodels (MRC, CLB and ADT) in each time step. [MeAsp] and [Ser] are MeAsp and Ser dynamic concentrations; u is the fluid velocity; $F_{run}$ and $T_{tumble}$ are the force and torque associated with direct run and tumble motion of an ECP.

Chemoattractant concentration distributions, however, are not static in real systems. As the ECP moves, it would disturb the flow and chemoattractant concentration fields. The proposed coupled MRC-CLB-ADT model accounts for spatiotemporal variations in chemoattractant concentrations due to ECP-fluid interactions. At each time step, (i) the CLB calculates the fluid velocity field, u, in response to ECP motion, and passes the computed fluid velocity field to the ADT; (ii) the ADT updates the concentration fields for MeAsp and Ser, [MeAsp] and [Ser] respectively, based on the new fluid velocity field and then sends the updated concentration fields to the MRC; and (iii) the MRC calculates the ECP motility in the fluidic environment with the new concentration fields and gradients. The MRC passes the running force, $F_{run}$, and tumbling torque, $T_{tumble}$, to the CLB in the subsequent time step to calculate angular and translation velocities of the ECP, the new position of the ECP, the new position of the receptor cluster on the ECP’s surface, and the resultant local disturbances in the flow field due to ECP motion. Figure 5 displays the flow of information in the MRC-CLB-ADT model for simulations discussed in Section 3.2.

### 2.5. Simulation Parameters

The motility of chemosensory particles in a fluidic environment is typically described by Reynolds number, $Re=|U_{p}|D_{p}/\tilde{\nu }$ where $D_{p}$ is the particle size. In our simulations, considering the average $|U_{p}|$ being $6.09\times 10^{-3}$ cm/s, $\tilde{\nu }$ being $8.70\times 10^{-3}$ cm${}^{2}/$s, and the representative size of the ECP being 2 cm, the Re associated with the ECP motility is 1.4, residing in the creeping flow regime. In this case, the ECP travels from one side of the square flow domain of 3232.4 cm${}^{2}$ to the opposite side in a straight flow path in 2.6 h at a rate of 0.3% of its body length per second.

ECP’s mass was 4.2 g in simulations. The force strength associated with the run motion of ECP was set to $3.25\times 10^{-4}$ kN to keep Re on the order of 1. The time-scaling factor associated with tumbling-induced rotation of bacteria, Υ, in Equation (17), was previously estimated to be 5 from simulations with a single chemotactic particle released into a confined domain in the absence of inline obstacles [21]. Therefore, Υ = 5 was adopted in simulations in this paper.

In simulations, $|r_{pw}|$ = 1.5 lattice unit (l.u.) and ψ = 1. ECP-wall steric interaction forces would be non-zero only when surface boundary nodes of the ECP move within 1.5 l.u. (0.43 cm) of stationary wall surfaces to avoid physically unrealistic overlaps [36]. When the boundary nodes of ECP the moves in within 1.5 l.u. of wall nodes, instantaneous, short-lived (within a few time-steps) relatively large (typically within an order of magnitude of $F_{r_{b}}$) steric pulse applies to keep the ECP-wall separation distance larger than 1.5 l.u.

Our simulations were conducted with two unmixed chemoattractants, MeAsp and Ser. When these concentrations were imposed in the environment using the Equations (6) and (7), the scaling parameters ω and ν controlled the peak concentrations. ω=1 returned the optimal sensitivity of chemoreceptors to MeAsp in the imposed environment as observed in [28]. Two values of the scaling parameter ν (0.1 or 0.001) were chosen to control the sensitivity of the chemoreceptor response to Ser. When ν=0.1, a dense level of cell accumulation to the Ser peak was expected in [28] due to a higher peak concentration compared to the one with ν=0.001. Therefore, in our simulations, ω was kept at 1 while ν=0.1 or ν=0.001 to compare the transient behavior of ECPs in different scenarios given the imposed environment (i.e., Equations (6) and (7) were used instead of the ADT model) or the dynamic environment (i.e., the ADT module was coupled as shown in Figure 5).

In our simulations, the diffusion coefficient values, *D*, for Ser and MeAsp were chosen such that they are within the range of experimentally reported values for Tar and Tsr substrates [49,50,51]. Because diffusion of chemoattractants would be enhanced by disturbances (additional mixing) in the fluid [52] by ECP motion, the values of *D* were increased by a factor of 10 to account for enhanced diffusion in MRC-CLB-ADT simulations. The factor of 10 was chosen so that the concentration distributions were spreading out reasonably within 50,000 time steps. However, the actual value of the enhancement factor and *D* could be obtained from experiments in future studies. Thus, for our simulations, *D* for Ser is set to $8.7\times 10^{-5}$ cm${}^{2}$/s and *D* for MeAsp is set to be 1.08 times higher than for Ser.

The scaling parameter *r* in Equations (6) and (7), is set to 14.3 cm. The initial maximum concentrations of MeAsp and Ser are located at (14.3 cm, 28.9 cm) and (42.9 cm, 28.9 cm), respectively, which correspond to $P_{1}=\left(50,101\right)$ and $P_{2}=\left(150,101\right)$ on a lattice grid. The minimum concentrations for MeAsp and Ser, $C_{a0}$ and $C_{s0}$, are set to 0.1 μM. In our simulations, 1 unit of concentration corresponds to 1 μM.

## 3. Results

In MRC-CLB-ADT simulations discussed in the subsequent sections, except for the validation test (Appendix A.1) and supplementary simulations (Appendix A.2) in Appendix, the fluid was initially quiescent and the fluid domain was bounded in all directions. A no-slip (wall) condition was imposed along the domain boundaries when the flow domain was bounded. Simulation results were reported at dimensionless times (i.e., in LB units) to ease the repeatability of the results, but simulation times can be expressed in seconds by multiplying their dimensionless counterparts by a factor of 0.938. Similarly, spatial lengths in LB units can be expressed in cm by multiplying them by a factor of 0.286. The MRC-CLB-ADT model was coded in MATLAB [53].

### 3.1. Simulations with Imposed Temporally-Invariant, Spatially-Variant Chemoattractant Concentrations

**Figure 6:**
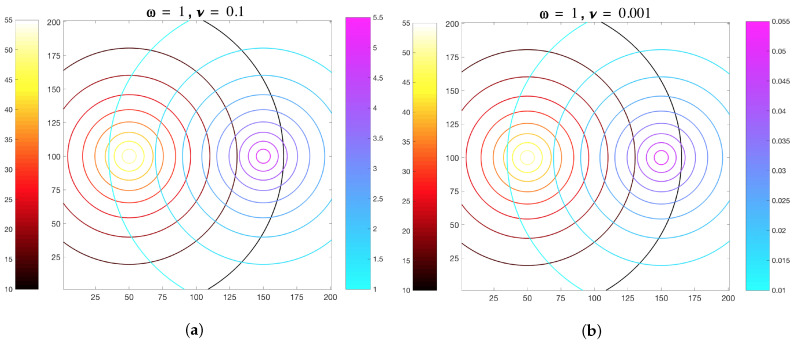
Spatially-variant, temporally-invariant MeAsp and Ser concentration fields in a 2D fluidic domain. Axes represent distances across the domain and colorbars represent amino acid chemoattractant concentrations in ▵C, (red/yellow = MeAsp, leftward side of gradient and blue/magenta = Ser, rightward side of gradient). In MRC-CLB simulations, two different ratios of chemoattractant gradient were chosen, with MeAsp gradient parameter ω set at ω = 1, and Ser gradient parameter ν set at either (**a**) ν = 0.1 or (**b**) ν = 0.001.

Base case simulations involving temporally-invariant, spatially-variant chemoattractant concentration fields were used to compare MRC-CLB model results to the simulation results by Edginton and Tindall [28]. Time-invariant chemoattractant concentration fields (Figure 6) computed by Equations (6) and (7) were imposed onto the flow field, instead of being computed in each time-step by the ADT model. Hence, temporal disturbances in the chemoattractant concentration fields due to motility of the ECP were not accounted for. These base case simulations are classified as “Imposed” due to the static nature of the chemoattractant concentration fields artificially imposed onto the fluid domain.

The MeAsp gradient parameter was set to ω = 1, based on previously reported Tar sensitivity curve data in [54]. The gradient parameter for Ser was set to ν = 0.1 or ν = 0.001. These ν values, in combination with the fixed ω gradient value, were chosen to account for changes in the bias of an ECP to travel toward increasing MeAsp concentrations due to saturation effects [28,54]. To account for the biased motion of an ECP in this environment, ten trial simulations were performed with ν = 0.1 or ν = 0.001. The ECP trajectories were different in these replicates due to the randomness in the MRC method (Equation (17)) that would determine if the ECP would run or tumble. The set up for simulation was a bounded domain of size [1200]×[1200] in LB units, corresponding to 56.85 cm × 56.85 cm. In each replicate, an ECP was released from the center of the domain located at (101,101).

The coordinates (x,y) of the centroid of a motile ECP were tracked for 50,000 time steps, corresponding to 13 h. At any given time-step, if x>100, the ECP would be located in the right half of the domain, where the center of the Ser concentration field was initialized at $\left(150,101\right)$, which will be referred to as “On Ser Half”. Similarly, if x<100, the ECP would be located in the left half of the domain, where the center of the MeAsp concentration field was initialized at $\left(50,101\right)$, which will be referred to as “On MeAsp Half” hereafter.

**Figure 7:**
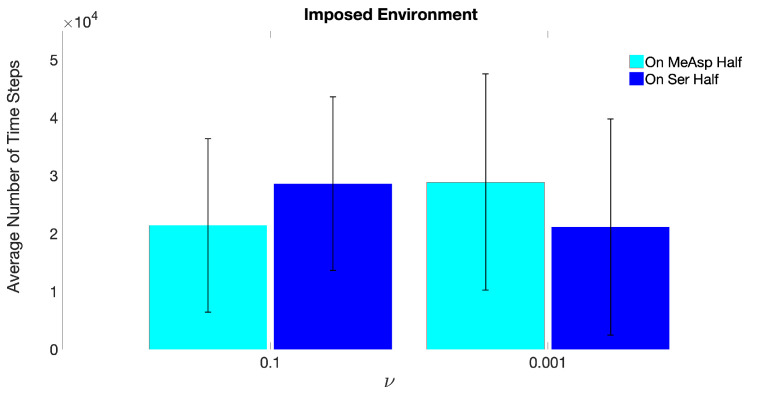
Average number of time steps the ECP resided in the “MeAsp half” or in the “On Ser Half”, concluded from ten replicates of MRC-CLB simulations with “Imposed” chemoatrractant concentration fields at the end of 50,000 time steps. Simulations were performed for two different values of ν (MeAsp parameter ω is fixed at ω=1, leading to ω/ν ratios of 1/0.1 and 1/0.001).

Figure 7 shows the *average number of time steps* from ten replicate simulations, for which the ECP was found in either the Ser- or MeAsp-half of the domain, in response to static chemoattractant gradients specified by ν = 0.1 or ν = 0.001. The large error bars indicate that trajectories of the ECP from these replicates were broadly distributed across the domain. The overall trends of the average values and error bars in Figure 7 are in good agreement with the results by [28], which showed an affinity of the chemosensory particle to (i) the Ser concentration field with scaling parameters ω=1 and ν=0.1, and (ii) the MeAsp concentration field with scaling parameters ω=1 and ν=0.001. These results reveal that ECP motility is governed by receptor sensitivity rather than absolute chemoattractant concentrations when time-invariant concentration fields are assumed.

### 3.2. Simulations with Spatiotemporal Variations in Chemoattractant Concentrations Computed via ADT Model

**Figure 8:**
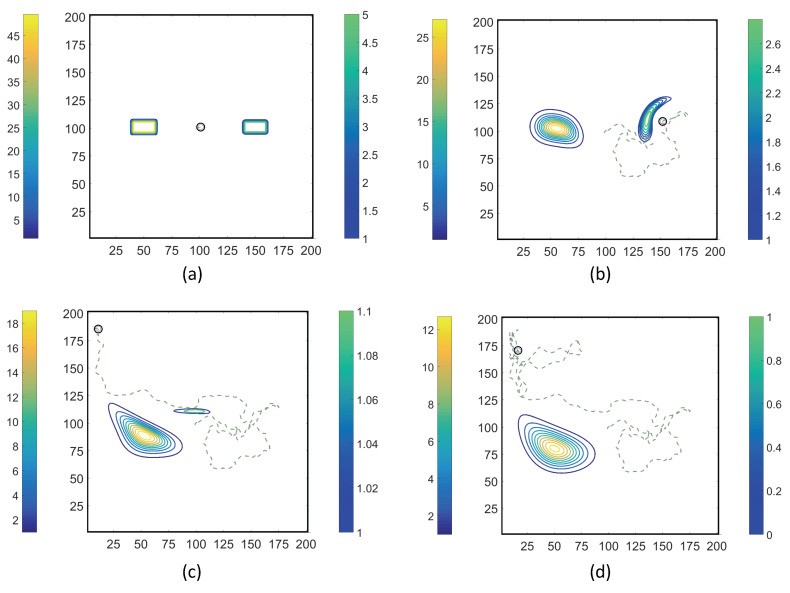
Trajectories of the ECP computed by the MRC-CLB-ADT model for Case 1 “ADT Point: Initial” and ν=0.1 at the dimensionless times (in LB units) of 10,000; 18,000; 30,000; and 50,000 are shown in (**a**–**d**). Simulation times can be expressed in seconds by multiplying the dimensionless times by a factor of 0.938. Each snapshot shows the contour plots of the concentration fields of MeAsp (left color bar) and Ser (right color bar). The center of MeAsp was initially on the left half and the center of Ser was on the right half of the domain. The total mass of MeAsp and Ser remained unchanged.

**Figure 9:**
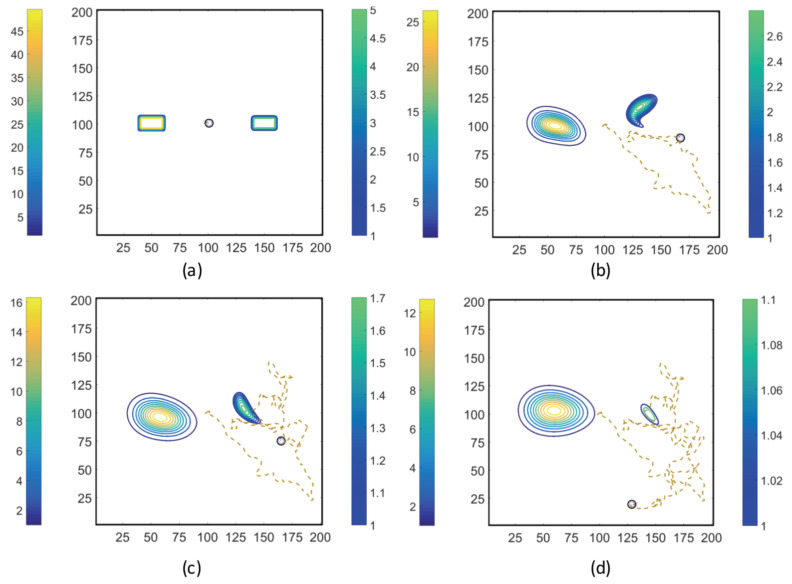
Trajectories of the ECP computed by the MRC-CLB-ADT model for Case 2 “ADT Point: Continuous” and ν=0.1 at the dimensionless times (in LB units) of 10,000; 18,000; 30,000; and 50,000 are shown in (**a**–**d**). Simulation times can be expressed in seconds by multiplying the dimensionless times by a factor of 0.938. Each snapshot shows the contour plots of the concentration fields of MeAsp (left color bar) and Ser (right color bar). The center of MeAsp was initially on the left half and the center of Ser was on the right half of the domain.

**Figure 10:**
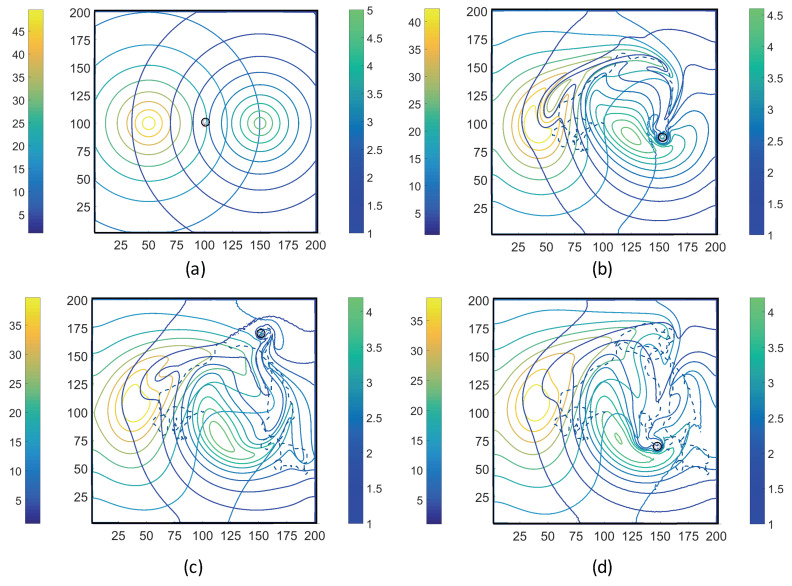
Trajectories of the ECP computed by the MRC-CLB-ADT model for Case 3 “ADT Imposed: Initial” and ν=0.1 at the dimensionless times (in LB units) of 10,000; 18,000; 30,000; and 50,000 are shown in (**a**–**d**). Simulation times can be expressed in seconds by multiplying the dimensionless times by a factor of 0.938. Each snapshot shows the contour plots of the concentration fields of MeAsp (left color bar) and Ser (right color bar). The center of MeAsp was initially on the left half and the center of Ser was on the right half of the domain.

**Figure 11:**
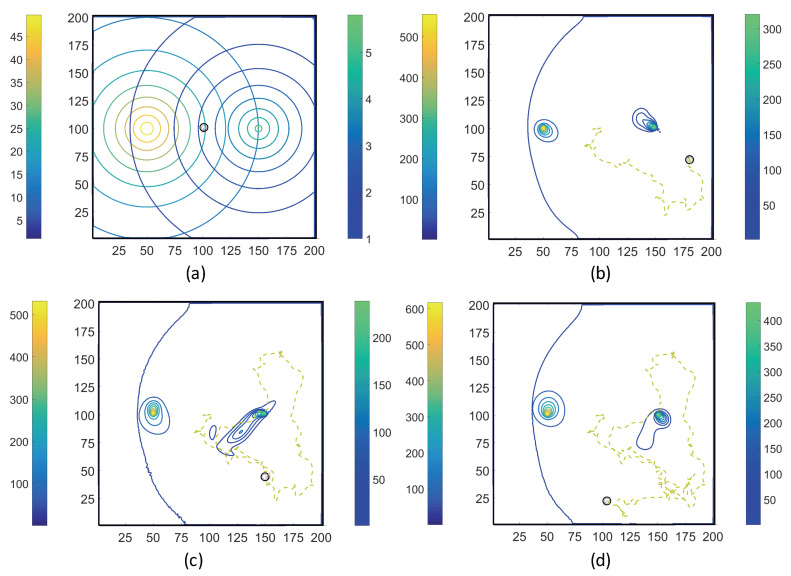
Trajectories of the ECP computed by the MRC-CLB-ADT model for Case 4 “ADT Imposed: Continuous” and ν=0.1 at the dimensionless times (in LB units) of 10,000; 18,000; 30,000; and 50,000 are shown in (**a**–**d**). Simulation times can be expressed in seconds by multiplying the dimensionless times by a factor of 0.938. Each snapshot shows the contour plots of the concentration fields of MeAsp (left color bar) and Ser (right color bar). The center of MeAsp was initially on the left half and the center of Ser was on the right half of the domain.

Using the coupled MRC-CLB-ADT model (Figure 5), two-dimensional simulations of ECP motility with incrementally improved realism in the problem set-up are discussed in this section. In order to represent the chemotatic behavior of an ECP in fluidic environments more realistically, the ADT model is used to simulate spatiotemporal variations in Ser and MeAsp concentration fields as the fluid is continuously disturbed by ECP motion. This is a significant conceptual and modeling improvement over the simulations discussed in Section 3.1, in which externally-computed time-invariant chemoattractant concentrations were artificially imposed onto the fluidic environment. Here, four cases with different chemoattractant source specifications were implemented in MRC-CLB-ADT simulations to analyze the effect of the initial distributions and release modes of chemoattractants on the transient chemotactic motility of an ECP:

- Case 1. “*ADT Point: Initial*”: At t=0, MeAsp and Ser were released into the fluid from point sources at $\left(x=50,y=101\right)$ and $\left(x=150,y=101\right)$, respectively. No additional chemoattractant releases occurred for t>0. Snapshots from this simulation are shown in Figure 8.
- Case 2. “*ADT Point: Continuous*”: After the initial condition was set up as in Case 1, $\left(▵C\right)$ of each chemoattractant was released into the fluid in each time-step for t>▵t from their respective point source locations, at which their maximum concentrations were maintained throughout the simulation. Snapshots from this simulation are shown in Figure 9.
- Case 3. “*ADT Imposed: Initial*”: Initial concentration fields of the chemoattractants were specified via Equations (6) and (7) and imposed onto the fluidic domain. No additional chemoattractant releases occurred for t>0. Snapshots from this simulation are shown in Figure 10.
- Case 4. “*ADT Imposed: Continuous*”: After the initial concentration fields of chemoattractants were established as in Case 3, $\left(▵C\right)$ of each chemoattractant was released into the fluid in each time-step for t>▵t from their respective point source locations. Snapshots from this simulation are shown in Figure 11.

Figure 8, Figure 9, Figure 10 and Figure 11 show trajectories of the ECP and temporal variations in concentration fields of the chemoattractants for ν=0.1. The fluid was quiescent in the beginning of the simulations. In early times, the ECP moved toward the Ser field on the right half of the fluidic domain. However, as the Ser concentration gradually diffused out, the trajectory of the ECP became unpredictable. The motile ECP continuously disturbed and altered the flow field and concentration fields of the chemoattractants as it exchanged momentum with the bulk fluid. The resultant alterations in the concentration fields subsequently affected the signaling pathway of the ECP, and hence, its tumbling and running motion. These simulations demonstrate that trajectories of the ECP were sensitive not only to initial distributions of the chemoattractants, but also to temporal variations in the chemoattractant concentration fields. Therefore, the consideration of ECP motion-induced disturbances in the fluid velocity field is imperative in ECP motility studies, and hence, should be accommodated in simulations.

**Figure 12:**
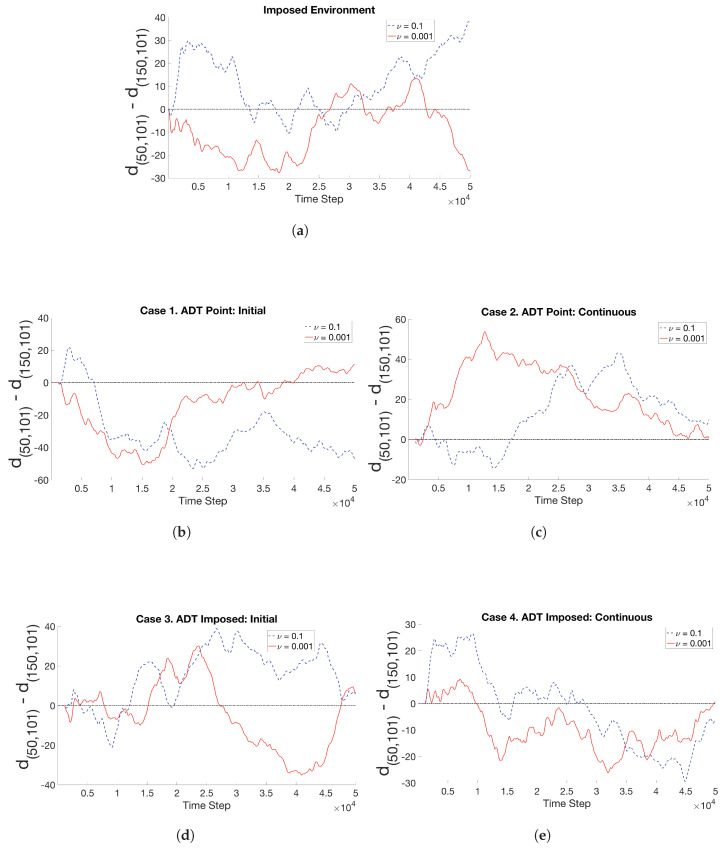
The source location of MeAsp and Ser is initially at $P_{1}=\left(50,101\right)$ and $P_{2}=\left(150,101\right)$, respectively. For ten replicates per simulation type, the time history of the average spatial distance between the position of the ECP and $P_{1}$ or $P_{2}$ is d${}_{\left(50,101\right)}$ or d${}_{\left(150,101\right)}$ in two different fluidic environments, characterized by ν= 0.1 or ν= 0.001. If d${}_{\left(50,101\right)}-$d${}_{\left(150,101\right)}<0$, the ECP retains its chemotactic activities mostly in the left half; otherwise, it would largely reside in the right half of the fluidic domain. The simulation types include (**a**) imposed concentrations, (**b**) Case 1, (**c**) Case 2, (**d**) Case 3, and (**e**) Case 4.

Ensemble-average of temporal changes in chemotactic activities of the ECP over ten replicates for Case 1 through Case 4 are shown in Figure 12. The ECP was released from the center of the bounded domain and its position was tracked for 50,000 time steps. In Figure 12, the distance between the ECP’s location at any given time-step and the center (50,101) of the MeAsp field at t=0 was computed and then averaged over ten replicates. The ensemble-average distance is denoted by $d_{\left(50,101\right)}$. Similarly, the distance between the location of the ECP at any given time-step and the center (150,101) of the Ser field at t=0 was computed and then averaged over ten replicates. The ensemble-average distance is denoted by $d_{\left(150,101\right)}$. Initially, the release locations or spatial distributions of the chemoattractants and the release locations of the ECPs were symmetric about the midpoint of the fluidic domain. Therefore, in each time-step, if [d${}_{\left(50,101\right)}-$d${}_{\left(150,101\right)}]<0$, the ECP would be on the left half of the domain, where the center of the MeAsp concentration field was initialized (“On MeAsp half”); otherwise it would be on the right half of the domain, where the center of the Ser concentration field was initialized (“On Ser half”).

The “Imposed” case in Figure 12a exhibits consistent results with the the bar graph in Figure 7 in the sense that the ECP motility was biased toward the MeAsp/left half for ν=0.001 (solid red curve), but toward the Ser/right half for ν=0.1 (dashed blue curve). Figure 12a further reveals that in the end of the simulations, the ECP tends to remain on the side (either Ser or MeAsp) from which it was initially released. The early steep rise for ν = 0.1 is associated with the strong initial response of the Tsr chemosensory component to the static Ser gradient. The initial average ECP behavior in the “Imposed” condition with ν = 0.001 also shows a strong tendency toward the MeAsp gradient, albeit with a lower sensitivity toward this attractant. This could be due to the lower sensitivity of the Tar receptor relative to that of the Ser receptor Tsr. Intermittent periodicity in ECP’s ensemble-average behavior in Figure 12a is associated with the sequence of its tumble and run motion. As the ECP continually runs and tumbles, it would move into, through, and out of zones of maximal chemoattractant concentration in some time steps. When the ECP senses continual temporal decreases in chemoattractant concentrations where it resides in the fluidic environment, it would begin to tumble and run back up to the zones with steeper concentration gradients. Based on the average positions of the ECP in reference to the center of the chemoattractant field in Figure 12a, such correction occurs sooner with Ser chemosensing for ν = 0.1 than MeAsp sensing with parameter ν = 0.001.

Simulations with ν = 0.1 and ν = 0.001 showed in Figure 12b–d that the ECP in general traveled across both halves of the fluidic domain, rather than residing mostly in one half the domain as in Figure 12a, when spatiotemporal disturbances in the concentration fields due to ECP motion are accounted for. Simulations with Case 1 (Figure 12b) or Case 4 (Figure 12e) show that the ECP maintained its transient chemotactic activities predominantly on the MeAsp half, as in the “Imposed” Case, when ν=0.001. On the other hand, the effect of this enforced biased travel on the ECP’s ensemble-average trajectories was negligible in Case 2 (Figure 12c) The ECP maintained more than 99% of its chemotactic activities on the Ser half. Furthermore, the point injection of chemoattractants to the fluidic domain, in comparison to the imposed concentration fields at t=0, resulted in transient chemotactic activities of the ECP on the opposite halves of the fluidic domain for ν=0.1. Even in Case 3, where the initial concentration fields were set up as in the “Imposed” case, the concentration fields were spatially and temporally evolving due to ECP motion, which affected run and tumble motion and overall ensemble-average trajectories of ECP. Similarly, bias in ECP’s motility toward the Ser half introduced by ν=0.1 had insignificant effect on particle ensemble-average trajectories in Case 1 and Case 4 when the concentration fields are not static. As a result, the ECP retained most of its chemotactic activities in the MeAsp half. In contrast, the biased ECP traveled toward the Ser half as in the “Imposed” case.

In summary, Figure 12 demonstrates that the biased ECP motility in a fluidic environment with multiple static chemoattractant concentration fields (i.e., “Imposed” environment) can be enforced and controlled through a fixed decisive factor of ν. However, if the concentration fields dynamically evolve spatially and temporally, ECP motility and trajectories are determined by its interactions with dynamically-evolving surrounding fluidic and chemoattractant environments, which cannot be represented accurately by a static factor ν.

## 4. Discussion

**Figure 13:**
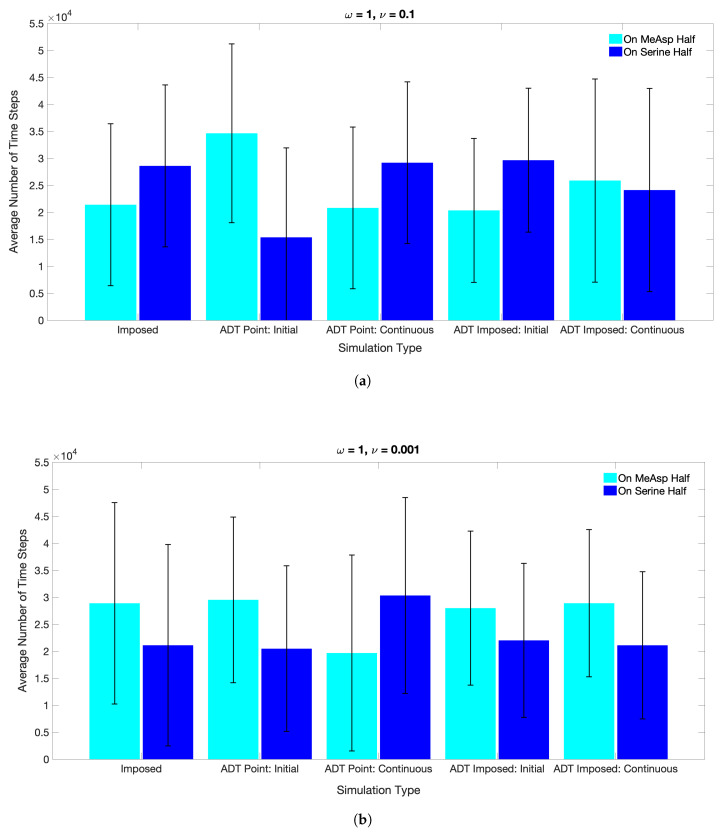
Ten simulations of 50,000 time steps each were performed for the“Imposed” case, and Case 1 through Case 4, in which trajectories of an ECP in a fluidic environment with an ω/ν ratio of (**a**) 1/0.1 and (**b**) 1/0.001 were traced. Heights of the bars correspond to the total residence time of an ECP either in the right half, in which the Ser concentration was initialized, or in the left half, in which the MeAsp concentration was initialized, of the fluidic domain. The first set of bars in both (**a**) and (**b**) are repeated from Figure 7 while the remaining sets are from ADT model simulations.

The average number of time steps the ECP spent in the MeAsp half or Serine half (i.e., the mean residence time of the ECP in each half) computed by the MRC-CLB-ADT model for Case 1 through Case 4 is compared against the “Imposed” case in Figure 13. Although ECP trajectories in Case 3 and the “Imposed” simulations were different in Figure 12a,d, the resultant mean residence times of the ECP and the associated errors were comparable in Figure 13. As noted previously, Case 3 and the “Imposed” had the same initial chemoattractant distributions, but the concentration field dynamically evolved in Case 3, unlike in the “Imposed” simulation . Although trajectories of the ECP were different in Case 3 and the “Imposed’ in each realization, the ensemble-average (the statistical mechanics) ECP motility data were similar and appear to be insensitive to spatiotemporal variations in the chemoattractant fields when the chemoattractants are spatially distributed initially and no additional chemoattractants are introduced into the fluid in later times. Hence, in such problem set-ups, the mathematically simpler and computationally less-expensive “Imposed” case could be used to evaluate the statistical mechanics of ECP motility in the design or performance assessment of ECPs.

When the chemoattractants were continuously released into the flow field in Case 4, the ECP preferentially spent more time in the MeAsp half, regardless of the value chosen for ν (Figure 13). This rather surprising result can be explained by the concentration distributions in Figure 11. Case 4 simulation is similar to the “Imposed” simulation with the exception that ▵C of the chemoattractants was continuously injected at the point sources in each time step. Although the injection maintained the highest local concentration of chemoattractants at the source locations, MeAsp spread more rapidly than Ser throughout the domain due to its higher diffusion coefficient, which in turn more strongly influenced the chemotactic activities of the ECP. This is clear from Figure 12e that shows a strong bias of ECP trajectory toward the MeAsp half over the entire simulation period.

When the chemoattractants were only initially released into the fluid as point sources in Case 1, the ECP spent more time in the MeAsp half of the domain. The maximum chemoattractant concentrations occurred at the point source location only at the start (Figure 10b). Because the ECP was initially farther away from point source locations and no additional chemoattractants were released into the fluid at later times, the ECP was incapable of accurately detecting the chemoattractants in early times. However, as advection and diffusion processes redistributed the chemoattractants as the time advanced, ECP trajectories were governed by continuously spreading and gradually diminishing local concentration gradients of chemoattractants.

When the chemoattractants were continuously released into the fluid for t>0 in Case 2, the ECP retained its chemotactic activities mostly in the Ser half, regardless of the value chosen for ν. A comparison of Case 1 and Case 2 simulations with ν = 0.001, (Figure 10b,c) shows that the ECP in Case 2 was initially positioned too far from MeAsp to detect any signal and respond to it. As a result, despite the smaller Ser gradient parameter value, the ECP responded to Ser only. Apparently, Tsr sensitivity dominated over Tar sensing even for the lower ν value. Similarly, ECP response to Ser in simulations with ν = 0.001 was delayed as the ECP was initially too far from the Ser point source location (Figure 10c, ν = 0.1). However, stronger Tsr response was observed after local chemoattractant gradients were established in the fluid and sensed by the ECP at approximately time step 15,000. These findings provide compelling evidence that unlike in Case 3, the “Imposed” condition would not be suitable to simulate chemotactic behaviors of the ECP in the problem set-ups in Case 1, Case 2, and Case 4.

In summary, unlike previously reported simulation results with imposed static chemoattractant concentration fields [28], our results with dynamically changing chemoattractant fields in response to ECP motion reveal for the first time that the relative magnitudes of ω and ν are not the sole factors in determining on which side of the fluidic domain an ECP would reside in. Instead, mutual dynamic interactions between particle motion and dynamically-varying concentration and flow fields would determine the statistical mechanics (ensemble-average) of ECP motility. Moreover, the release modes of the chemoattractants (point vs. non-point source and/or initial vs. continuous releases) and spatiotemporal evolution of chemoattractant concentration fields are found to be the critical factors for chemotactic activities and the statistical mechanics of motility of ECP, which would determine the zone(s) where an ECP would reside in a fludic domain.

## 5. Conclusions

We developed a new multiscale chemotactic motility model to investigate the behavior of ECPs in dynamic fluidic environments with spatially and temporally-varying gradients of two chemoattractants in response to ECP motility. We quantified the behavior of ECPs mimicking *E. coli* chemotaxis in fluidic environments containing the unmixed amino acid attractants *N*-methyl-L-aspartate and L-serine which function as strong chemical signals in the chemosensory system of *E. coli*. This was accomplished by formulating a novel dynamically coupled numerical model, MRC-CLB-ADT model, to simulate the motility of ECPs in an initially quiescent fluid with spatially and temporally-evolving chemoattractant concentrations. The MRC-CLB-ADT is capable of simulating the motility of ECPs by accommodating (i) spatial and temporal distributions of two distinct, non-interacting chemoattractants, (ii) effects of ECPs motion on the spatial and temporal distributions of the chemoattractants, and (iii) interactions of ECPs and the surrounding fluid environment.

**Figure A6:**
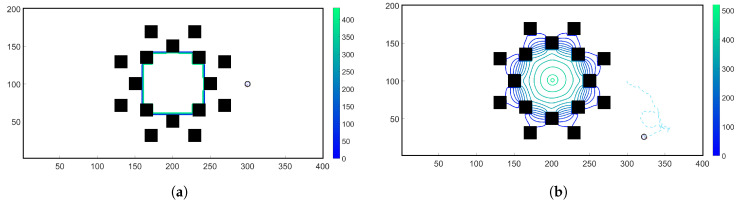

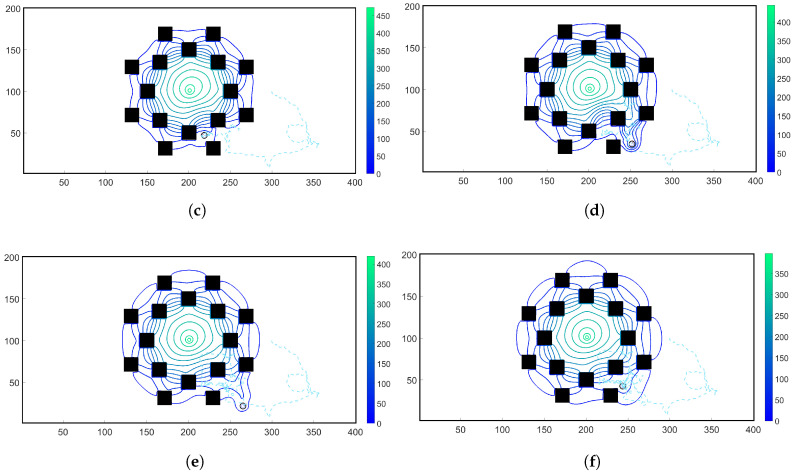
Snapshots of the chemoattractant concentration field (whose contour lines are shown by solid lines) and trajectory of a chemosensory particle (shown by a light blue dashed line) at time-steps of 1000; 14,000; 32,000; 42,000; 52,000; and 62,000 in (**a**) through (**f**), respectively. Because the chemoattractant was continuously added to the center of the domain, the total chemoattractant mass in the fluidic domain increased in time. As a result, the domain was saturated with the chemoattractant at later times, which led to the random walk in particle trajectory towards the end of the simulation.

The results and analysis of a variety of simulation set-ups allowed quantitative assessment for how chemoattractant distribution, particle-fluid dynamics, and particle-chemoattractant concentration field interactions affect the chemosensing properties of the ECPs. Results from “Imposed” simulations supported previous findings indicating that the ECP behavior is governed by receptor sensitivity rather than absolute attractant concentration [28,54]. Similarly, more recent biochemical work [55] has also substantiated the long-held notion that the networked architecture of chemosensory receptor arrays in *E. coli* plays a vital role in the cell’s robust response behavior. Results of simulations that incorporate CLB-ADT emphasize the importance of advective-diffusive transport phenomena in modeling ECP trajectories in fluidic environments. Fluid effects on ECP chemosensing are significant both in those environments with pre-established chemoattractant gradients and in environments with distal point source chemoattractants. An example is shown in Appendix A.3: Figure A6 and represent a more complex environment with solid obstacles and one attractant. ECPs will behave differently depending on whether or not chemoattractant is continually introduced into the environment. Further development and refinement of the model will be valuable for the exploration of other aspects of fluid-based ECP motility, such as the effects of fluid viscosity, non-Newtonian fluids, MCP stoichiometry in chemosensory arrays, chemoeffectors with varying affinities for MCPs, including repellent molecules, and environments comprising varying ECP population densities.

## Figures and Tables

**Figure A1:**
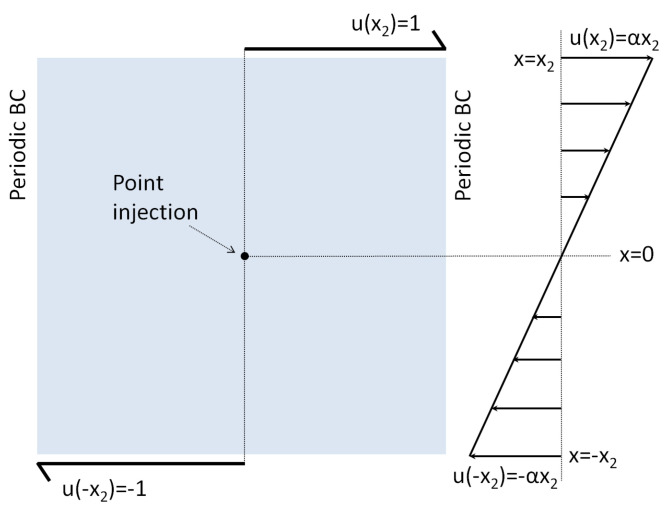
Benchmark problem used to validate the ADT model based on the LBM. Temporal and spatial distributions of a substrate were computed following its instantaneous injection into a Couette flow as a point source at x=0. The flow domain was sheared along the top and bottom boundaries with the same magnitude of velocity, *u*, but in the opposite directions at a vertical distance of $x_{2}$ from the injection point. The lateral boundaries are periodic. The velocity profile is shown on the right panel.

**Figure A2:**
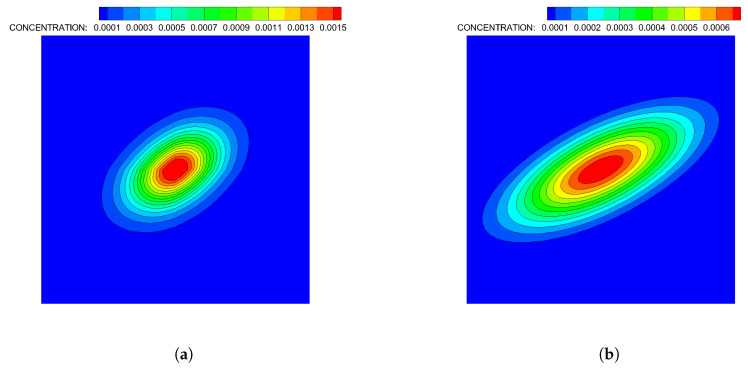

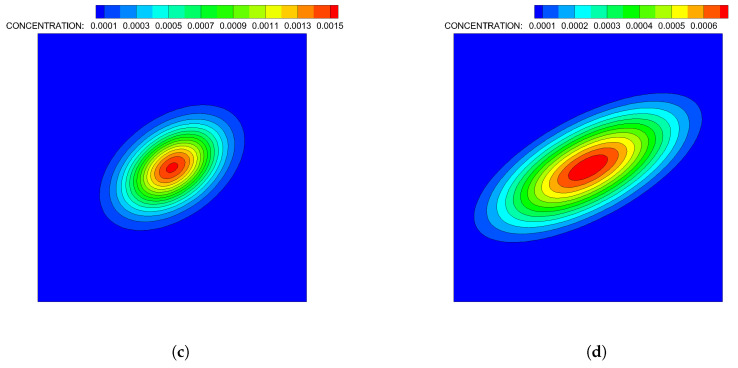
Comparison of analytically and numerically (by the ADT model) computed concentration gradient dynamics for solute following instantaneous release into a Coutte flow at center of domain. The ADT model simulation results at (**a**) $t=\tau_{s}$ and (**b**) $t=2\tau_{s}$; analytic results at (**c**) $t=\tau_{s}$ and (**d**) $t=2\tau_{s}$.

**Figure A3:**
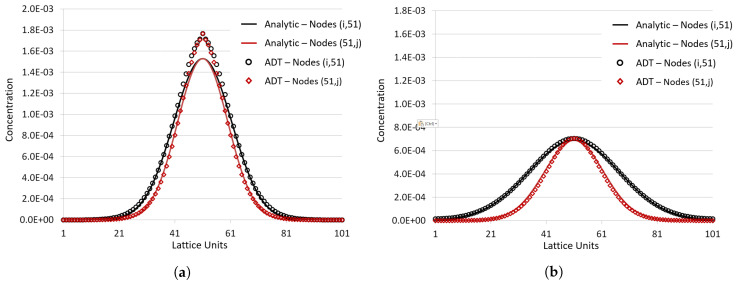
Comparison of analytically and numerically computed (by the ADT model) substrate concentration profiles along the horizontal and vertical cross-sections with respect to the center of the concentration field at (**a**) $t=\tau_{s}$ and (**b**) $t=2\tau_{s}$. $x_{c}=\left(x_{c},y_{c}\right)=\left(51,51\right)$ is the center point, where the instantaneous point source was located.

**Figure A4:**
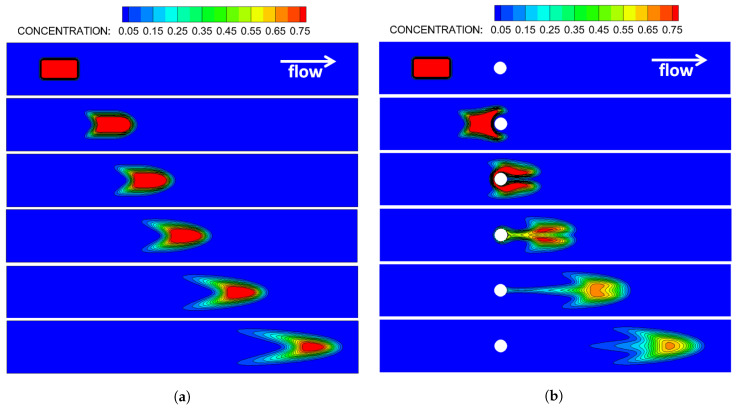
Advective and diffusive transport of a chemoattractant, characterized by Pe=500, in a horizontal flow channel (**a**) without internal obstacles or (**b**) with a circular obstacle shown in white. Snapshots are taken at at Ω= 0.0, 0.11, 0.18, 0.25, 0.35 and 0.49.

**Figure A5:**
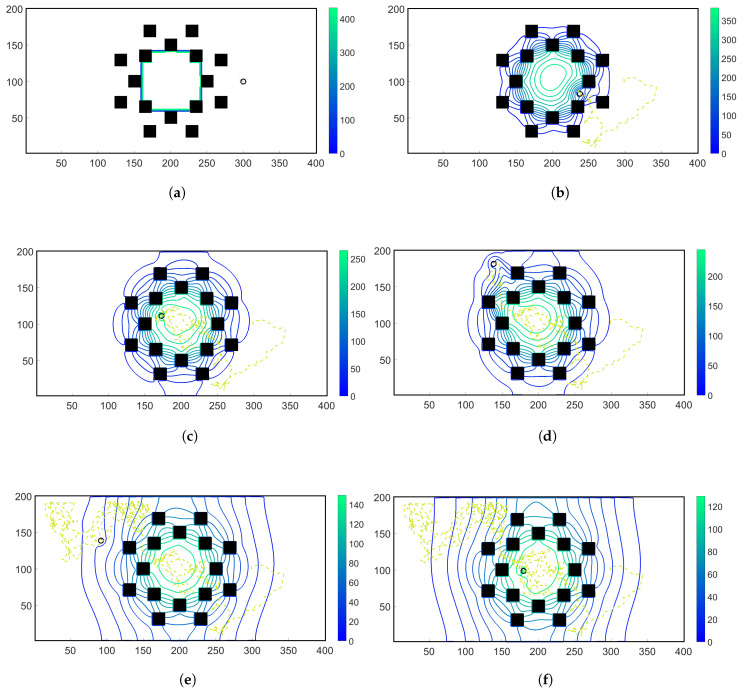
Snapshots of the chemoattractant concentration field (whose contour lines are shown by solid lines) and trajectory of a chemosensory particle (shown by a light green dashed line) at time-steps of 1000; 28,000; 68,000; 80,000; 174,000; and 204,000 in (**a**) through (**f**), respectively. The total chemoattractant mass remained constant throughout this simulation.

## Appendix A

### Appendix A.1. Numerical Validation of the ADT Model

Prior to coupling with the MRC and CLB models, the ADT model was validated using a two-dimensional analytic solution provided in [56]. The analytic solution calculates spatiotemporal evolution of an inert substrate after being instantaneously injected into a Coutte flow at the center, $x_{c}=\left(x_{c},y_{c}\right)$, of the flow domain. The concentration distributions across the flow domain are computed by

(A1) $$C\left(x,t\right)=\frac{exp\left[-\frac{1}{2}x^{T}\Upsilon^{-1}\left(t\right)x\right]}{2\pi \sqrt[{}]{det\left[\Upsilon \left(t\right)\right]}},$$

where x is the Cartesian coordinates of the lattice node, *t* is the time, $\Upsilon \left(t\right)$ is the variance matrix, det is the determinant operator. The variance matrix, $\Upsilon \left(t\right)$, is defined as

(A2) $$\Upsilon =\left[\begin{array}{cc} \Upsilon_{11} & \Upsilon_{12}\\ \Upsilon_{21} & \Upsilon_{22} \end{array}\right]=\left[\begin{array}{cc} 2D_{1}t+\frac{2}{3}D_{2}\alpha^{2}t^{3} & D_{2}\alpha t^{2}\\ D_{2}\alpha t^{2} & 2D_{2}t \end{array}\right],$$

where $D_{1}$ and $D_{2}$ are the diffusion coefficients and $\alpha =u_{x}\left(x_{2}\right)/x_{2}$, in which $u_{x}$ is the x-component of the specified fluid velocity at the top boundary and $x_{2}$ is the vertical distance between the substrate release location and the top boundary.

The benchmark problem is illustrated in Figure A1. The model parameters in the LBM simulation were left in non-dimensional lattice units to be consistent with the benchmark problem. The size of the flow domain was set to 100×100 (lattice node numbers run from 1 to 101 in both directions). The fluid across the flow domain was initially stationary (i.e., u=0). Specified fluid velocity was imposed at the top and bottom boundaries, but in the opposite directions. The lateral boundaries were assumed to be periodic. This resulted in zero flow velocity at the center of the flow domain (at $x_{c}=\left(x_{c},y_{c}\right)$). In the analytic solution, a point-like unit substrate concentration was instantaneously injected into the flow domain at the center, $x_{c}$. The diffusion coefficient, *D*, of the substrate was set to unity (D=1). The $D_{1}$ and $D_{2}$ in the variance matrix in Equation (A2) were also set to unity for an isotropic and homogeneous flow domain. From the half length of the domain, $x_{2}=50$, and the specified velocity at the top boundary, $u(x_{2})=$1, we compute $\alpha =2\times 10^{-2}$. Hence, the typical timescale associated with the shear rate is $\tau_{s}=\alpha^{-1}=50$.

Instantaneous point injection of the substrate in the analytic model [56] was represented by a point-like initial distribution of the substrate described by $C\left(x_{c},t=0\right)=\delta \left(x_{c}\right)$, for which C→∞ for t=0 in Equation (A1). We simulated instantaneous substrate injection as a point source at $x_{c}$ in the LBM by implementing $C\left(x_{c},t=0\right)=1$, which resulted in $\sum_{x}C\left(x,t\right)=C\left(x_{c},t=0\right)=1$ for t>0.

A comparison of the spatial distributions of the substrate computed by the analytic solution and numerical simulations via the ADT model for $t=\tau_{s}$ and $t=2\tau_{s}$ are shown in Figure A2 and Figure A3. Figure A2 shows that analytically and numerically computed spatial distributions and temporal evolution of the substrate are the same for $t=\tau_{s}$ and $t=2\tau_{s}$, except that the peak concentration at $x_{c}$ appears to be higher from the ADT model solution than the analytic solution at $t=0.2\tau_{s}$. This was further confirmed from the horizontal and vertical cross-sections of the concentration field with respect to the center of the flow domain in Figure A3. Use of $C\left(x_{c},t=0\right)=\delta \left(x_{c}\right)$ in the analytic model and $C\left(x_{c},t=0\right)=1$ in the ADT model led to discrepancies in the peak concentration, $C\left(x_{c},t>0\right)$ at early times while matching the concentration profiles almost perfectly away from the point source. At later times, concentration profiles computed by the ADT model matched the analytically computed concentrations perfectly. The total mass was strictly conserved in every time-step in the ADT model simulation.

### Appendix A.2. ADT Model Simulations of Advective-Diffusive Substrate Transport in a Flow Channel

Figure A4 demonstrates ADT model simulation of advective diffusive transport of a substrate (chemoattractant) in a horizontal flow channel with and without an internal obstacle. A no-slip boundary condition was implemented on the channel walls and a periodic boundary condition was imposed at the inlet and outlet. A steady Poiseuille flow was established in the horizontal channel prior to start of ADT model simulations. The Péclet number, $Pe=u_{ss}W/D$ was set to 500 in these simulations, in which $u_{s}$ is the average steady fluid velocity at the inlet prior to release of the chemoattractant into a flow domain and *W* is the channel width. The dimensionless time, Ω, was rendered as $\Omega =tu_{s}/L$, where *L* is the channel length and Ω=0 corresponds to the time at which chemoattractant was released into the steady flow field. τ=0.501 in these simulations. The results show that the obstacle enhances chemoattractant diffusion, which in turn alters its temporal and spatial gradients, which would have significant effects on motility behavior of a chemosensory particle.

### Appendix A.3. With Simulated ADT-Concentrations Moving around Obstacles

Figure A5 and Figure A6 demonstrate the motility of a chemosensory particle in an initially quiescent fluid with a dynamically evolving chemoattractant concentration field. The size of the domain was set to 200 × 400 in lattice units. The chemoattractant concentration was initialized at the center of the domain, surrounded by concentric rings of solid obstacles depicted by black squares. A no-slip boundary condition was imposed on the domain walls and on the surfaces of internal obstacles.

In Figure A5, the chemoattractant concentration was initialized at the center of the domain at t=0 with no additional chemoattractant releases for t>0. Hence, the total chemoattractant mass remained constant throughout the simulation. On the other hand, for the simulation results shown in Figure A6, the chemoattractant concentration was initialized at the center of the domain at t=0 and in each subsequent time-step, t>▵t, ▵C was released from the center of the flow domain.

In both simulations, the chemosensory particle, shown as a black circle, was initially placed outside the concentric ring of obstacles while the chemoattractant was initialized inside the ring. In MRC-CLB-ADT model simulations, the chemosensory particle sensed and avoided inline obstacles while navigating towards the spatially- and temporarily-varying maximum concentration gradients inside the ring. Consideration of the effects of inline obstacles on the spatial and temporal distributions of the chemoattractant concentration field, also simultaneously altered by particle-fluid hydrodynamic forces, is the unique modeling capability of the MRC-CLB-ADT model.

### Appendix A.4. Tables of Parameters and Variables for All the Modules

#### Appendix A.4.1. Prescribed Parameters for the MRC Module

**Table A1:** Descriptions and values of predetermined parameters in Equations (1)–(7).

Parameter	Description [Source]	Value
*N*	Number of chemoreceptors in receptor cluster [28]	18
$v_{a}:v_{s}$	Ratio of Tar to Tsr receptors [28]	1:1.4
$K_{a}^{on}$	Dissociation constant in the on state of Tar receptors [17]	0.012 μM
$K_{a}^{off}$	Dissociation constant in the off state of Tar receptors [17]	0.0017 μM
$K_{s}^{on}$	Dissociation constant in the on state of Tsr receptors [17]	10${}^{6}$ μM
$K_{s}^{off}$	Dissociation constant in the off state of Tsr receptors [17]	100 μM
${\left[CheR\right]}_{tot}$	Total CheR concentration [17]	0.16 μM
${\left[CheB\right]}_{tot}$	Total CheB concentration [17]	0.28 μM
${\left[CheZ\right]}_{tot}$	Total CheZ concentration [17]	*
$mb_{0}$	Basal motor bias [17]	0.65
*H*	Motor Hill coefficient [17]	10.3
*a*	Scaling factor for methylation [17]	0.0625
*b*	Scaling factor for demethylation [17]	0.0714
$k_{Z}$	Rate constant [17]	$30/{\left[CheZ\right]}_{tot}$μM${}^{-1}$$s^{-1}$
$k_{Y}$	Rate constant [17]	100 μM${}^{-1}$ $s^{-1}$
$k_{s}$	Scaling coefficient [17]	0.45μM
$\gamma_{Y}$	Rate constant [17]	0.1 s${}^{-1}$
$C_{a0}$	Minimum chemoattractant concentration for MeAsp	0.1 μM
$C_{s0}$	Minimum chemoattractant concentration for Ser	0.1 μM
ω	Scaling parameter for MeAsp gradient	1
ν	Scaling parameter for Ser gradient	0.1 or 0.001
$\left(x_{a},y_{a}\right)$	Location of the maximum MeAsp concentration initially	(14.3 cm, 28.9 cm)
$\left(x_{s},y_{s}\right)$	Location of the maximum Ser concentration initially	(42.9 cm, 28.9 cm)
$L^{*}$	Domain length	57 cm
*r*	Scaling parameter for domain size	14.3 cm

l(*) indicates that the total [*CheZ*]*_tot_* concentration does not need to be specified explicitly since it is canceled with itself when multiplying with *k_Z_* in Equation (3).

**Table A2:** Descriptions of functions and variables in Equations (1)–(7).

Variable	Description [Source]
*F*	Total free energy differences between ‘on’ or ‘off’ state [28]
h(m)	Offset energy given by 1−m/2 [28]
[MeAsp]	Chemoattractant MeAsp concentration [28]
[Ser]	Chemoattractant Ser concentration [28]
$A_{c}$	Probability of the cluster activity [17]
[CheY-P]	Concentration of phosphorylated CheY [17]
*m*	Receptor methylation [17]
mb	Motor bias [17]
x,y	Horizontal and vertical coordinates

#### Appendix A.4.2. System Parameters and Variables for the CLB ad ADT Modules

**Table A3:** Notations used in CLB and ADT Modules. FFS and PFS correspond to the Fluid Flow Submodule (Section 2.2.1) and the Particle Flow Submodule (Section 2.2.2) of the CLB Module.

Notation	Type	Description	Used by
$c_{s}$	parameter	speed of sound	FFS, ADT
e	parameter	unit velocity vectors	FFS, PFS , ADT
$f_{i}$	variable	population densities associated with fluid flow	FFS, PFS
$f_{i}^{eq}$	variable	equilibrium distribution associated fluid flow	FFS
$f_{m}$	parameter	particle force strength	PFS
g	parameter	acceleration due to external forces	FFS, PFS
$g_{i}$	variable	population densities associated with substrate transport	ADT
$g_{i}^{eq}$	variable	equilibrium distribution associated substrate transport	ADT
*i*	variable	index	FFS, PFS, ADT
$m_{p}$	parameter	particle mass	PFS
r	variable	position vector	FFS, PFS, ADT
$r_{b}$	variable	position of boundary nodes of ECP	PFS
$r_{b}^{c}$	variable	covered lattice nodes by ECP motion	PFS
$r_{b}^{u}$	variable	uncovered lattice nodes by ECP motion	PFS
$r_{c}$	variable	position of the ECP’s centroid	PFS
$r_{cl}$	variable	location of the cluster receptor	PFS
$r_{i}$	variable	distance vector	PFS
$|r_{it}|$	parameter	repulsive threshold distance	PFS
$r_{p}$	parameter	particle radius	PFS
$r_{pw}$	variable	surface to surface distance between the wall and ECP	PFS
$r_{v}$	variable	position of intra-particle lattice node	PFS
*t*	variable	time	FFS, PFS, ADT
$t^{*}$	variable	post-collision time	PFS
u	variable	fluid velocity	FFS, PFS, ADT
*C*	variable	chemoattractant concentration	ADT
*D*	parameter	diffusion coefficient of chemoatractant	ADT
$F_{run}$	variable	forces associated with running motion of ECP	PFS
$F_{r_{b}}$	variable	hydrodynamic forces	PFS
$F_{r_{b}}^{c,u}$	variable	forces associated with (un)covered lattice nodes	PFS
$F_{r_{p}w}$	variable	steric interaction forces between ECP and wall	PFS
$F_{T}$	variable	total force imposed on ECP	PFS
$I_{p}$	parameter	moment of inertia of ECP	PFS
$L^{*}$	parameter	domain length	FFS
*M*	parameter	Mach number	FFS
$T_{tumble}$	variable	torque associated with tumble motion of ECP	PFS
$U_{p}$	variable	translation velocity of of ECP	PFS
$\theta_{cl}$	variable	rotation angle of the receptor cluster	PFS
$\tilde{\nu }$	parameter	fluid kinematic viscosity	FFS
ρ	variable	fluid density	FFS, PFS
τ	parameter	relaxation parameter associated fluid flow	FFS
$\tau_{c}$	parameter	relaxation parameter associated substrate transport	PFS
ψ	parameter	stiffness parameter associated with steric interaction forces	PFS
$\omega_{i}$	parameter	weights associated with the D2Q9 lattice geometry	FFS, PFS, ADT
$\tilde{\varphi }$	variable	uniform deviate	PFS
▵P	parameter	pressure differential	FFS
▵t	parameter	temporal increment	FFS, PFS, ADT
▵x	parameter	lattice spacing	FFS, PFS, ADT
▵θ	variable	angular rotation of ECP	FFS
$\Omega_{tumble}$	variable	angular velocity of ECP due to its tumbling motion only	PFS
$\Omega_{p}$	variable	angular velocity of of ECP	PFS
Υ	parameter	time-scale factor associated with ECP’s angular rotation	PFS
